# In sickness and health: Effects of gut microbial metabolites on human physiology

**DOI:** 10.1371/journal.ppat.1008370

**Published:** 2020-04-09

**Authors:** Robert W. P. Glowacki, Eric C. Martens

**Affiliations:** Department of Microbiology and Immunology, University of Michigan Medical School, Ann Arbor, Michigan, United States of America; University of Wisconsin Medical School, UNITED STATES

The connection between intestinal microbes and human health has been appreciated since the 1880s with Theodor Escherich’s investigation of *Escherichia coli* and other fecal bacteria. Escherich hypothesized that indigenous micoorganisms play roles in both digestion and intestinal diseases [1]. In the last century, our understanding of the bacteria, viruses, archaea, and eukaryotes that normally inhabit the gut has expanded alongside the rest of the field of microbiology, and numerous fundamental roles have been established for this community, now termed the microbiome. As speculated by Escherich, these roles definitively include nutrient digestion [2, 3] and protection from invading pathogens [4] but also extend to short- and long-term instruction of the immune system [5–7] and production of a wide range of metabolites that are unable to be produced by human physiology. Although the gut microbiome is typically described as being composed of nonharmful or beneficial microorganisms, it is now appreciated that both individual species [8] or multiple community members acting together [9, 10] can exert pathogenic effects, which are often more subtle than those of classical pathogens. Indeed, the presence of common intestinal microorganisms with discrete virulence factors (e.g., enterotoxins, genotoxins) that may only manifest in diseases like colorectal cancer or inflammatory bowel disease (IBD) over long periods of time or in certain host genetic backgrounds obscures the definition of pathogen.

Accelerated in the 2000s by the “-omics” revolution, along with a recent resurgence of cultivation [11–13], countless studies in the past 2 decades have implied or established connections between altered gut microbiomes and many diseases. These studies have demonstrated the malleability (or fragility) of the microbiome in the face of environmental and dietary perturbations encompassing antibiotic use [14], geography [15], immigration [16], and dietary changes, including fiber deprivation [17, 18]. Although Escherich’s original ideas were logically predicted with respect to microbiome effects in the gut, less-anticipated connections between gut microbes and health have extended to neurobiology [19–22] and systemic immune responses that impact allergy [23]. Emerging studies, often extending from omics-based observations, are providing causal and mechanistic understanding of the relationships that connect host responses with changes in the microbiome and its metabolism. Here, we look at recent examples that illustrate how the gut microbiome can augment or perturb host physiology through complementary or novel metabolism often initiating or modifying disease trajectories. The studies we highlight provide details that underscore the importance of gut microbes in human health, which Escherich postulated long ago.

## The impact of gut bacterial metabolites on host physiology

**Fig 1:**
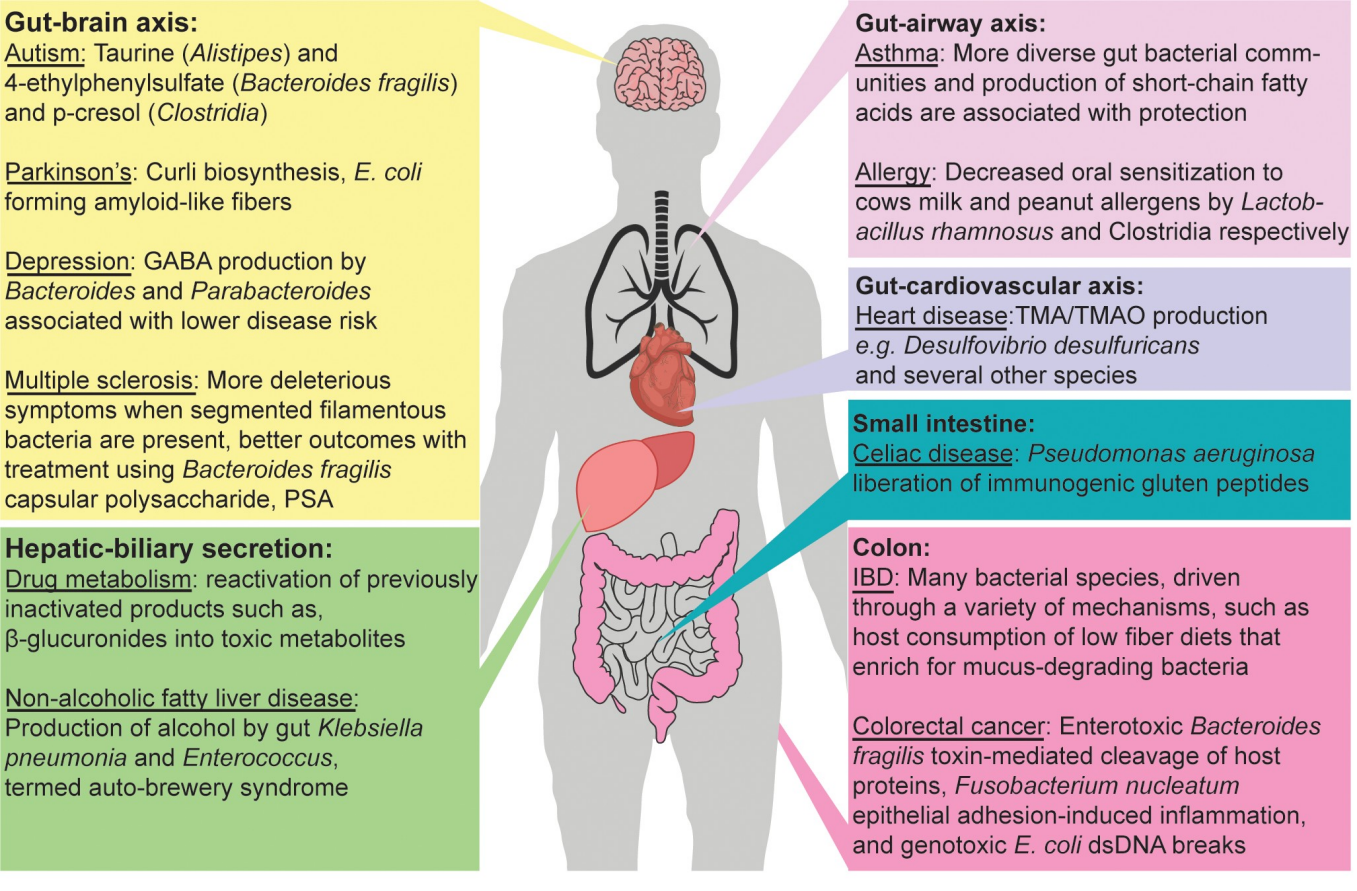
Effects of the gut microbiome on host health. Some of the many known effects of the gut microbiome on diseases of various organ systems are illustrated. References for associations highlighted in the figure that are not mentioned in detail in the main text: autism [21, 33], Parkinson’s [64, 65], depression [29], multiple sclerosis [66], drug metabolism [54, 55], nonalcoholic fatty liver disease [67, 68], asthma [69], allergy [70, 71], heart disease [51], Celiac disease [72], IBD [27], and colorectal cancer [73–76]. dsDNA, double stranded DNA; GABA, gamma-aminobutyric acid; *E*. *coli*, *Escherichia coli*; IBD, inflammatory bowel disease (a collection of several intestinal disorders that includes Crohn’s disease and ulcerative colitis); PSA, capsular polysaccharide A from *Bacteroides fragilis*; TMA/TMAO, trimethylamine/ trimethylamine N-oxide.

The collective diversity of microbial species that compose the gut microbiome harbor approximately 10 million unique, annotated genes [24]—probably many more [25]—that are not present in the human genome. Through our individual microbiomes, each of us has a personalized subset of this gene repertoire that substantially exceeds the genes in our human genome. With this unique genetic potential, our microbiomes are equipped to produce an astonishing array of microbiome-produced products (MPPs): metabolites and other cellular products like polysaccharides and curli fibers, which, in many cases, do not remain confined to the gut. The impacts of specific MPPs and the presence/absence of individual species/strains that produce them have been implicated in a wide range of diseases both in the gastrointestinal tract and beyond (**Fig 1**). Effects in the gut include preventing pathogen invasion through bile salt modifications [26], mucus layer erosion when the host lacks dietary fiber [27], and accelerating DNA damage that promotes tumor formation [9, 10]. More surprisingly, studies have drawn connections to neurological conditions such as Parkinson’s disease (PD) [22, 28], depression [29, 30], and autism spectrum disorder (ASD) [31–33], suggesting that certain bacteria and their MPPs (e.g., curli fibers in PD; the metabolites, 4-ethylphenylsulfate, *p*-cresol, taurine, and 5-aminovaleric acid in ASD) can contribute to these states (**Fig 1**).

Although a variety of host pattern recognition receptors (PRRs) directly sense microbial “danger” signals, including those from microbiome symbionts, emerging evidence also supports the idea that we interact directly with microbes through additional cellular receptors. A recent study using a forward chemical genetics screen showed that MPPs from several dozen bacteria promote direct interactions with G-protein coupled receptors (GPCRs), a broad class of receptors important for physiological responses spanning mood regulation, immune function, and intestinal peristalsis [34]. This included a strain of *Morganella morganii* that converts L-phenylalanine into phenethylamine, a psychoactive compound that can be fatal in individuals taking monoamine oxidase inhibitor drugs [34]. Studies have also shown that the bacteria that convert tryptophan to tryptamine stimulate the colonic-restricted GPCR, 5HT_4_R, resulting in increased intestinal transit time [35]. Additionally, bacterial production of *N*-acyl amides regulates glucose homeostasis and possibly appetite [36]. MPPs also interact with other receptors, such as the aryl-hydrocarbon receptor (AhR). For example, production of AhR ligands, such as indole 3-aldehyde by *Lactobacillus reuteri*, leads to increased IL-22 production and a mucosal immune response against *Candida albicans* [37]. Just as the effects of potentially pathogenic bacteria can be altered because of the content of pathogenicity islands and other accessory gene content, studies like the ones noted previously often reveal variable effects from strains of the same species. This is an important consideration when formulating potential probiotics or other live bacterial therapeutics. A recent example of this is the implication of *L*. *reuteri* (strain SP-C2-NAJ0070) as an exacerbator of systemic lupus erythematosus (SLE) symptoms in a TLR7-dependent manner, an effect that is not attributable to other *Lactobacilli* [38].

Another class of molecules, which have previously been well-studied in pathogenic bacteria, the cyclic di- and trinucleotides (CDNs/CTNs), are also emerging as molecules that interact with host sensors. The structural diversity of these compounds has expanded from purine-based to include pyrimidine-based molecules [39]. Although not definitively linked to aspects of host health and disease, some of these CDNs can activate host immune pathways through PRRs, such as stimulator of interferon genes (STING) and reductase-controlling NF-ĸB (RECON) protein. Homologs of CDN synthesis operons are widespread in both commensal and pathogenic bacteria, including the prevalent *Bacteroides* genus. A recent study suggests that bacteria have evolved new ways of evading/enhancing host PRR recognition through synthesis of unique CTNs or modified CDNs not efficiently sensed by host PRRs [39].

A final group of MPPs that are just beginning to be explored are bacterial capsular polysaccharides (CPS), which are enriched and highly diversified in several lineages of gut bacteria [40]. For example, just 14 sequenced strains of the common gram-negative symbiont *Bacteroides thetiotaomicron* harbor 47 different configurations of gene clusters for producing CPS [41]. Expression of some of these CPS alters the way this bacterium is sampled by macrophages and presented to T cells [42]. A subset of zwitterionic CPS, first discovered in *Bacteroides fragilis* but present in other species, has immunomodulatory properties, as do CPS and extracellular polysaccharides produced by members of different phyla, the Actinobacteria [43], Proteobacteria [44], and Firmicutes [45, 46]. These bacterial surface coatings are likely to be under intense pressure to diversify their glycan structures, perhaps to evade host immune responses, bacteriophages, and microbe-mediated killing. In the process, they have fortuitously synthesized chemical structures that interact with the host epithelium and immune system (**Fig 1**), providing additional advantages during colonization and also opportunities for researchers to exploit these molecules for potential drug development [47].

Collectively, the studies highlighted previously illustrate how host cells have evolved to sense and interact with a variety of metabolites or products that are uniquely microbial, which is the basis of much innate immune recognition and of central importance in the tolerance of the dense human gut microbiome [48]. Better understanding of these interactions may prove helpful in leveraging these existing chemical relationships to design new drugs that alter immune responses or other aspects of host cellular biology.

## Metabolism of drugs and other xenobiotics by gut microbes

Just as members of the microbiome produce novel molecules that interact with human physiology, they also have the capacity to modify exogenous chemicals (xenobiotics), many of which are the drugs used to treat diseases. Two prominent examples are inactivation of the cardiac drug digoxin by *Eggerthella lenta* [49] and related plant-derived cardenolides [50] and the ability of several species to convert the common dietary compound choline to trimethylamine (TMA), which is subsequently converted by the host to harmful trimethylamine-*N*-oxide (TMAO), which promotes cardiovascular disease (**Fig 1**) [51–53]. Another process that has been characterized mechanistically is drug reactivation following β-glucuronic acid conjugation in the liver and biliary secretion back into the gut. This process is catalyzed by gut bacterial β-glucuronidases, which are widely present in gut bacteria [54] and have broad substrate specificities [55, 56] that allow them to reactivate toxic drugs like the chemotherapeutic irinotecan. This process may be circumvented by drugs that, in turn block, β-glucuronidases to halt drug retoxification.

A recent example highlights how the common gut commensal *Bacteroides thetaiotaomicron* and related Bacteroidetes metabolize a range of xenobiotics using previously undescribed mechanisms. One of these involves degradation of the nucleoside-based antiviral drugs brivudine and sorivudine to the hepatotoxic compound bromovinyluracil (BVU) through the action of a nucleoside phosphorylase [57]. Homologs of this gene are found in many members of the phylum, suggesting that toxic BVU could accumulate at faster rates based on which members of the microbiota are present or their abundance. Another study expanded the repertoire of drugs that can be metabolized by *B*. *thetaiotaomicron*, identifying 18 drugs that are modified by an additional 17 unique enzymes [58]. Further highlighting that multiple bacteria can work synergistically in the gut, a recent study discovered a pathway for enzymatic inactivation of the Parkinson’s drug, levodopa (L-dopa). This stepwise mechanism involves *Enterococcus faecalis*, which first decarboxylates L-dopa to active dopamine, followed by a dehydroxylase from *E*. *lenta* that inactivates L-dopa and produces *m-*tyramine [59]. These studies point to variations in the gut microbiome as often overlooked reasons why therapy fails, or patients have intolerable side-effects to treatments. Thus, the gut microbiome is another factor to consider during treatment of disease, which may eventually require integration of both microbiome sequencing and culture/biochemistry-based approaches.

Beyond commensal bacteria altering the effects of therapeutic drugs, recent studies involving *Clostridium difficile* (*Cd*) have potentially uncovered a link as to why patients taking calcium supplements, proton-pump inhibitors, and nonsteroidal anti-inflammatory drugs (NSAIDs) may be predisposed to infection or have more severe outcome. The germination signal for *Cd* is known to be intestinal bile salts, such as taurocholate, with glycine acting as a co-germinant. However, recent studies in vitro [60] and in vivo [61] have identified a role for Ca^2+^, which circumvents the requirement for glycine and could be derived from dietary supplements or increased during malabsorption. The connection between Ca^2+^ and *Cd* germination suggests a plausible mechanism for why individuals with high intestinal Ca^2+^ due to diet or poor absorption due to proton-pump inhibitors or low vitamin D are at greater risk of *C*. *difficile* infection (CDI). NSAIDs were recently shown to alter the community structure of the microbiota, potentially creating an environment in which CDI is more severe [62]. Although the study only examined responses to the NSAID indomethacin, dysregulation of intestinal tight junctions was observed leading to more severe disease through translocation of *Cd* across the epithelium.

Some of the findings described previously can be leveraged to design tools to guide drug selection and therapeutic interventions. A recently developed in silico tool is being used to model interactions between drug classes and bacterial enzymes with activities against these drugs [63]. This approach was used to successfully predict 3 previously unknown xenobiotic metabolic pathways by gut microbes that were confirmed through in vitro studies [63]. As knowledge of microbiome–drug interactions expands, it is likely that future personalized medicine approaches will use these predictive tools coupled with in vitro and in vivo models to guide treatment regimes in a myriad of diseases.

## A way forward in the search for better therapeutics

From the studies highlighted here, the concepts of commensal and mutualistic bacteria always being “neutral” or “beneficial” to host biology is almost certainly naive. Rather, commensals, and even mutualists, may exhibit pathogenic activities, albeit in more subtle ways. Whereas true pathogens are equipped with toxins and machinery that directly damages cells, our more numerous, nonpathogenic symbionts may not be as directly insidious. The means by which these commensal organisms exhibit pathogenic tendencies are contextually dependent on factors such as diet, host genetics, drug intake, and production of MPPs. Furthermore, when considering whether the presence of a species could be beneficial or detrimental based on metagenomic or 16S ribosomal DNA sequencing approaches, the unique accessory genome of each strain, and not just phylogeny, needs to be considered. The context-specific activities of our common symbiotic bacteria may have both transient (acute) and chronic (long-term) health effects that likely influence disease states across organ systems. Leveraging the results of functional studies that link the microbiome to these diseases will illuminate new paths to manipulate the potential deleterious effects of the gut microbiome on health.
